# Effects of vaccination against COVID-19 on overactive bladder symptoms on young population

**DOI:** 10.3389/fmed.2024.1338317

**Published:** 2024-06-25

**Authors:** Marta de-la-Plaza-San-Frutos, Elisa García-García, Beatriz Martínez-Pascual, Isabel Mínguez Esteban, Diego Domínguez-Balmaseda, M. Dolores Sosa-Reina

**Affiliations:** ^1^Faculty of Sport Sciences, Universidad Europea de Madrid, Madrid, Spain; ^2^Research Group on Exercise Therapy and Functional Rehabilitation, Faculty of Sport Sciences, Universidad Europea de Madrid, Madrid, Spain; ^3^Woman & Health Research Group, Department of Physiotherapy, Faculty of Sport Sciences, Universidad Europea de Madrid, Madrid, Spain; ^4^Musculoskeletal Pain and Motor Control Research Group, Faculty of Sport Sciences, Universidad Europea de Madrid, Madrid, Spain; ^5^Masmicrobiota Group, Faculty of Health Sciences, Universidad Europea de Madrid, Madrid, Spain; ^6^Real Madrid Graduate School, Faculty of Sports Sciences, Universidad Europea de Madrid, Madrid, Spain

**Keywords:** COVID-19, vaccinations, overactive bladder, lower urinary tract, OABSS

## Abstract

**Introduction:**

The vaccines developed against COVID-19 have different modes of action, with a primary focus on the spike protein of the virus. Adverse effects following vaccination have been reported, including local and systemic symptoms. Understanding the potential side effects on the urinary tract after vaccination is of importance. Actively investigating and comprehending the potential impact on the urinary tract, we can enhance public health strategies and pave the way for safer and more effective vaccination programs.

**Methodology:**

The study was based on an online survey that included the Spanish Version of the Overactive Bladder Symptom Score (OABSS-S); 2,362 men and women replied to the survey. After the application of the exclusion criteria, 1,563 participants were insured. In the context of COVID-19, individuals were questioned regarding several key factors related to their vaccination status and medical history. These factors included the number of vaccine doses received, the specific type of vaccine administered, whether they had previously contracted COVID-19, and the frequency of prior infections, if applicable.

**Results:**

A total of 1,563 (74.7% women and 27.3% men) subjects between the ages of 18 and 45 completed the survey and were included in the final analyses. The most frequently administered vaccine type was Pfizer-BioNTech (42.2%), and most subjects received three doses. The proportion of females who received the AstraZeneca vaccine and do not require to urinate during the night is significantly higher compared to males (59.1% vs. 33.3%; *p*<0.05). The proportion of individuals who urinate five or more times during the night is higher in those who have received a single vaccine dose than in those who have received three doses (2.2% vs. 0.1%; *p*<0.05).

**Conclusion:**

COVID-19 vaccination has been found to impact the lower urinary tract (LUT) and overactive bladder (OAB). Initially, LUT symptoms worsened, and OABSS-S scores increased after the first vaccine dose in individuals under 45 years old. However, symptoms improved after receiving the third and fourth doses. Gender differences were observed in the vaccination effects. Men vaccinated with AstraZeneca reported a higher number of nighttime voids, while women vaccinated with Moderna reported more daytime voids.

## 1 Introduction

Although COVID-19 had a substantial impact on demographic indicators during the first 2 years of the pandemic, the overall global health progress over the 72-year period evaluated has been profound, with considerable improvements in mortality and life expectancy, largely due to vaccines.

Vaccines against COVID 19 will go down in history as the greatest scientific and logistical effort by pharmaceutical companies (1). In January 2020, the phase I clinical trial of the first vaccine against the new coronavirus began (2). In late 2020, the first SARS-CoV-2 vaccine was approved by the European Medicines Agency (EMA) and by the US Food and Drug Administration (FDA) from the Pfizer-BioNTech companies, followed by the mRNA vaccine developed by Moderna and the adenovirus-vectored vaccine from Oxford University/AstraZeneca (3). Vaccines against SARS-CoV-2 have different modes of action (4). One focus of the vaccines that have been developed is directed against the S protein or spike on the surface of the coronavirus. Protein S is the protein on the virus that binds to the cellular receptor, human ECA2 (ACE2), and triggers infection. Acting on this key entry point is key to developing the protective response against SARS-CoV-2 (5). Many questions have been raised about vaccination that are being answered as time goes on, such as the adverse effects caused by vaccination (6). Self-reported adverse effects following COVID-19 vaccination have been described in several studies with mRNA-based (Moderna and Pfizer-BioNTech) and viral vector (AstraZenko) vaccines, the most common being localized symptoms at the first dose, such as pain at the injection site, and systemic symptomatology such as weakness, headache, fever, chills and fatigue at the second dose (7, 8). Several studies report an increase in lower urinary tract urological symptomatology such as increased frequency and urgency of urination (9–11). Such is the case of research published on 889 subjects where lower urinary tract and overactive bladder symptomatology was studied before and after vaccination. This cross-sectional study used the overactive bladder symptom score (OABSS), and its results indicate that up to 13.4% experienced a worsening of storage after vaccination (9). This may be due to the immune inflammation response following vaccination, as in the case of cardiac tissue, where myocardial and pericardial inflammation have been reported (12). The mechanism of inflammation in vaccine-induced myocarditis is unknown, but it has been linked to the active component of the vaccine, the mRNA sequence encoding the spike protein, the immune response following vaccination, over-activation of cytosines and the active ingredients of the vaccine (12, 13).

The need for studies that reveal more information on the effect of vaccination in other systems such as the lower urinary tract is of scientific interest, as well as its connections with the vaccination doses received and type of vaccine. The objective of this study will be to determine the relationship or association that exists after vaccination against COVID-19 and the sequelae of the lower urinary tract as a secondary effect in young population.

The selection of subjects aged between 18 and 45 is due to the fact that older individuals have a higher risk of developing urinary tract pathologies due to factors such as muscle weakening, hormonal changes, prostate enlargement, chronic diseases, immobility, and increased susceptibility to infections.

The research aims to address gaps in the scientific literature related to the effects of COVID-19 vaccination on the lower urinary tract (LUT) and overactive bladder (OAB), particularly in individuals under 45 years of age, after vaccination. It seeks to better understand the relationship between the vaccine-induced immune response, based on the type and number of vaccine doses received, as well as differences between men and women.

## 2 Materials and methods

The present study follows the STROBE (14) guideline for an observational, descriptive, cross-sectional model. It was approved by the Ethics Committee from the Universidad Europea de Madrid (CIPI/22.247). In addition, all the participants answered an online survey after the acceptance of an informed consent form. The data has been adhered to the ethical standards of the Declaration of Helsinki (15) and the 3/2018 law for Personal Data Protection.

### 2.1 Participants

The total sample recruited was 2,362 subjects. The sample was recruited on healthcare facilities, community centers, online platforms, and professional networks. After the application of the exclusion criteria, 1,563 participants were included. The inclusion criteria were: (a) People between 18 and 45 years; (b) with at least one dose of any vaccine against the COVID-19; (c) Speak or understand Spanish fluently. Exclusion criteria: (a) History of pelvic and/or urologic surgery; (b) History of use of pharmacology for micturition control such as anticholinergic drugs or diuretics; (c) Oncological processes; (d) Pregnancy; (e) Postpartum of less than 1 year after partum; (f) BMI equal or greater than 30; (g) Diabetes; (h) History of bladder stones; (i) Neurological disorders or alterations; (j) Motor disorders or alterations that prevent the autonomy to move and move around; (k) Cardiovascular disease; (l) Chronic pelvic Pain; (m) Interstitial cystitis.

### 2.2 Outcome measures

For data collection purposes, an online survey was designed. Prior to commencing the survey, participants were provided with an introduction explaining the study’s purpose, followed by the requirement to provide informed consent by signing a consent form. The survey consisted of 17 questions were the Spanish Version of the Overactive Bladder Symptom Score (OABSS-S) was included, the OABSS-S evaluates four main symptoms of overactive bladder: urinary frequency, urinary urgency, urgency incontinence, and nocturia (16). The rest of the questionnaire was completed with self-designed questions about inclusion and exclusion criteria. Participants were also queried regarding the number of vaccine doses received, the specific type of vaccine administered, and their history of COVID-19 infection, including the number of times they had experienced it.

### 2.3 Sample size and statistical analysis

For the calculation of the sample size, a 15% difference in proportions was taken into account (9). Accordingly, the sample size formula for the difference in proportions was employed, with a significance level set at 5%: $n=\frac{{\left(Z_{\alpha /2}\;\sqrt{2}\right)}^{2}\left(p_{1}\left(1-p_{1}\right)+p_{2}\;\left(1-p_{2}\right)\right)}{{\left(p_{1}-p_{2}\right)}^{2}}$ . Consequently, an approximate sample size of 1,483.

Descriptive statistics were performed for presenting the demographic variables and urinary-related adverse effects after COVID-19 vaccination prevalence using absolute frequencies (*n*) and relative frequencies (%). All of the comparisons were conducted according to type of vaccine and number of vaccines administered.

Chi^2^ test and comparison of proportions with Bonferroni adjust was used to study the association between type of vaccine as well as number of vaccines administered and the urinary-Related adverse effects after COVID-19 Vaccination. *P*-values ≤0.05 were considered statistically significant. Statistical analyses were conducted using a commercial statistical software (Statistical Package for the Social Sciences, version 29.0; SPSS, Inc., Armonk, NY, USA).

## 3 Results

### 3.1 Demographic characteristics

A total of 1,563 subjects between the ages of 18 and 45 completed the survey and were included in the final analyses. The baseline characteristics of all included subjects are shown in Table 1. The majority (74.7%) of the subjects were women. The most frequently administered vaccine type was Pfizer-BioNTech (42.2%), and most subjects received three doses. A higher proportion of women have received a combination of vaccines compared to men (39.9% vs. 29.9%; *p* < 0.05), while a higher proportion of men have received the Pfizer-BioNTech vaccine compared to women (48.4% vs. 40.2%; *p* < 0.05). Additionally, a higher proportion of men have received two doses compared to women (45.3% vs. 34.2%; *p* < 0.05), while more women have received 3 and 4 doses compared to men (53.5% vs. 43.5% and 7.1% vs. 3.5%; *p* < 0.05).

**Table 1:** Demographic characteristics on included subjects.

Characteristics		Subjects included (*n* = 1,563)
Sex	Female, *n*, %	1,168 (74.7%)
	Male *n*, %	395 (25.3%)
Type of vaccine	AstraZeneca, *n*, %	90 (5.8%)
	Combination of vaccines, *n*, %	584 (37.4%)
	Moderna, *n*, %	229 (14.7%)
	Pfizer-BioNTech, *n*, %	660 (42.2%)
Number of vaccinations (Doses received)	1, *n*, %	90 (5.8%)
	2, *n*, %	579 (37.0%)
	3, *n*, %	797 (51%)
	4 or more, *n*, %	97 (6.2%)

### 3.2 Urinary adverse effects following COVID-19 vaccination

#### 3.2.1 Vaccine type

Upon considering all the subjects included in this study, no statistically significant differences were observed based on the type of vaccine received (Table 2). However, upon classifying the subjects according to their sex, some differences were noted. The impact of vaccine type on urinary symptoms can be visualized in the bar chart provided in Appendix 1.

**Table 2:** Urinary-related adverse effects after COVID-19 vaccination according to type of vaccine.

		Type of vaccine			
		AstraZeneca *n*, %	Moderna *n*, %	Pfizer-BioNTech *n*, %	Combination of vaccines *n*, %
Frequency of urination during the day *n* (%)	More than once every hour	10 (11.1%)	101 (17.4%)	129 (16.2%)	8 (8.2%)
	Every 1–2 h	25 (27.8%)	165 (28.5%)	220 (27.6%)	27 (27.8)
	Every 2–3 h	40 (44.4%)	204 (35.2%)	295 (37%)	44 (45.4%)
	Every 3–4 h	11 (12.2%)	81 (14.0%)	130 (16.3)	13 (13.4%)
	Less than once every 4 h	4 (4.4%)	28 (48%)	23 (2.9%)	5 (5.2%)
Number of times of urination during the night *n* (%)	None	50 (55.6%)	319 (55.1%)	482 (60.5%)	51 (52.6%)
	1 time	28 (31.1%)	195 (33.7%)	233 (29.2%)	37 (38.1%)
	2 times	7 (7.8%)	40 (6.9%)	55 (6.9%)	6 (6.2%)
	3 times	3 (3.3%)	15 (2.6%)	23 (2.9%)	1 (1%)
	4 times	0 (0%)	7 (1.2%)	3 (0.4%)	1 (1%)
	5 times or more	2 (2.2%)	3 (0.5%)	1 (0.1%)	1 (1%)
Reason for urinating *n* (%)	For convenience	5 (5.6%)	40 (6.9%)	72 (8.9%)	5 (5.2%)
	Minimal urgency	26 (28.9%)	220 (38%)	285 (35.8%)	47 (48.5%)
	Moderate urgency	51 (56.7%)	267 (46.1%)	381 (47.8%)	35 (36.1%)
	Several urgency	8 (8.9%)	47 (8.1%)	50 (6.3%)	9 (9.3%)
	Desperate urgency	0 (0%)	5 (0.9%)	10 (1.3%)	1 (1%)
Time Waited Before Urination *n* (%)	More than 60 min	23 (25.6%)	145 (25%)	235 (29.5%)	30 (30.9%)
	Between 30 and 60 min	24 (26.7%)	200 (34.5%)	299 (37.5%)	28 (28.9%)
	Between 10 and 30 min	28 (31.1%)	162 (28%)	184 (23.1%)	24 (24.7%)
	10 min or less	13 (14.4%)	64 (11.1%)	66 (8.3%)	14 (14.4%)
	Immediately	2 (2.2%)	8 (1.4%)	13 (1.6%)	1 (10%)
Frequency of Urgent Urination *n* (%)	Never	9 (10%)	81 (14%)	121 (15.2%)	16 (16.5%)
	Rarely	48 (53.3%)	297 (51.3%)	469 (58.8%)	54 (55.7%)
	A few times per month	17 (18.9%)	78 (13.5%)	110 (13.8%)	13 (13.4%)
	A few times per week	7 (7.8%)	59 (10.2%)	46 (5.8%)	7 (7.25)
	At least 1 time per day	9 (10%)	64 (11.1%)	51 (6.4%)	7 (7.2%)
Frequency of Inability to Reach the Toilet in Time Before Urination *n* (%)	Never	59 (65.6%)	374 (64.6%)	558 (70%)	62 (63.9%)
	Rarely	23 (25.6%)	159 (27.5%)	192 (24.1%)	32 (33%)
	A few times per month	6 (6.7%)	26 (4.5%)	31 (3.9%)	3 (3.1%)
	A few times per week	6 (6.7%)	26 (4.5%)	31 (3.9%)	3 (3.1%)
	At least 1 time per day	2 (2.2%)	17 (2.9%)	13 (1.6%)	0 (0%)

*Differences compared to 1 vaccine; †Differences compared to 2 vaccines; ‡Differences compared to three vaccines (*p* < 0.05) using Chi^2^ test and proportion comparison analysis with Bonferroni adjust.

##### 3.2.1.1 Results by sex

The following data presents the findings pertaining to the frequency of urination during the day, number of times of urination during the night, reason for urination, time waited before urination, frequency of urgent urination, and frequency of inability to reach the toilet in time before urination, categorized by gender.

###### 3.2.1.1.1 Frequency of urination during the day

No statistically significant differences were observed regarding the frequency of urination during the day between males and females according to the type of vaccine received.

###### 3.2.1.1.2 Number of times of urination during the night

Upon classifying subjects by sex, it was noted that the proportion of females who received the AstraZeneca vaccine and do not require to urinate during the night is significantly higher compared to males (59.1% vs. 33.3%; *p* < 0.05). Additionally, the proportion of men who received a combination of vaccines and had to urinate more than five times during the night is significantly higher compared to women (1.7% vs. 0.2%; *p* < 0.05).

###### 3.2.1.1.3 Reason for urination

No statistically significant differences were observed concerning the reason for urination between males and females or as per the type of vaccine received.

###### 3.2.1.1.4 Time waited before urination

Regarding subjects who received the Moderna vaccine, the proportion of men who can wait more than 60 min before urinating is significantly higher compared to women (40.3% vs. 25.2%; *p* < 0.05). Meanwhile, the proportion of women who can wait between 10 and 30 min before urinating is significantly higher compared to men (31.1% vs. 16.1%; *p* < 0.05).

###### 3.2.1.1.5 Frequency of urgent urination

No statistically significant differences were observed in the frequency with which study participants had to go to the toilet immediately between men and women according to the type of vaccine received.

###### 3.2.1.1.6 Frequency of inability to reach the toilet in time before urination

The proportion of men who received the Moderna and Pfizer vaccines or a combination of vaccines and had *“never”* failed to reach the toilet in time before urinating is significantly higher compared to women (82.3% vs. 56.9, 83.2% vs. 63.8, and 85.6% vs. 61.6%, respectively; *p* < 0.05). Similarly, the proportion of women who received the Moderna and Pfizer vaccines or a combination of vaccines and had “*rarely*” failed to reach the toilet in time before urinating is significantly higher compared to men (32% vs. 11, 37.1% vs. 11.5, and 27.1% vs. 11.5%, respectively; *p* < 0.05).

#### 3.2.2 Number of vaccine doses

Table 2 presents data on urinary-related adverse effects following COVID-19 vaccination, classified by vaccine type.

##### 3.2.2.1 Frequency of urination during the day

There were no statistically significant differences in the frequency of micturition during the day based on the number of vaccine doses received. No statistically significant differences were observed between sexes.

##### 3.2.2.2 Number of times of urination during the night

Among all study participants, the results indicated that the proportion of individuals who urinate five or more times during the night is higher in those who have received a single vaccine dose than in those who have received three doses (2.2% vs. 0.1%; *p* < 0.05). However, no statistically significant differences were observed regarding other number of doses.

Regarding sex of the participants, the proportion of women who have received three doses of the vaccine and urinate twice during the night is higher compared to men (7.8% vs. 3.5%; *p* < 0.05). Conversely, the proportion of men who have received four doses and urinate twice is higher than that of women (21.4% vs. 3.6%; *p* < 0.06).

##### 3.2.2.3 Reason for urinating

With respect to the reason for urinating, the proportion of people who urinate due to minimal urgency is higher in those who have received four doses of the vaccine than in those who have received one dose (48.5% vs. 28.9%; *p* < 0.05). Conversely, the proportion of individuals urinating due to moderate urgency is higher in those who have received a single dose compared to those who have received four doses (56.7% vs. 36.1%; *p* < 0.05). However, no statistically significant differences were observed based on sex of the participants.

##### 3.2.2.4 Time waited before urination

No statistically significant differences were observed in terms of the time that can be waited before going to the toilet. In terms of the sex of the participants, the proportion of men who received one dose of the vaccine and can wait for 30–60 min is significantly higher compared to women (40% vs. 20%; *p* < 0.05). Additionally, the proportion of women who received three doses and can wait for 10 min or less is significantly higher compared to men (16.9% vs. 14.7%; *p* < 0.05).

##### 3.2.2.5 Frequency of urgent urination

The proportion of individuals who must go to the toilet immediately with a frequency of “rarely” is higher in those who have received three doses of the vaccine compared to those who have received two doses (58.8% vs. 51.3%; *p* < 0.05). In contrast, the proportion of individuals who must go to the toilet immediately “a few times per week” or “once a day” is higher in those who have received two doses compared to those who have received three doses (10.2% vs. 5.8% and 11.1% vs. 6.4%, respectively; *p* < 0.05). No statistically significant differences were observed based on sex of the participants.

##### 3.2.2.6 Frequency of inability to reach the toilet in time before urination

**Figure 1:**
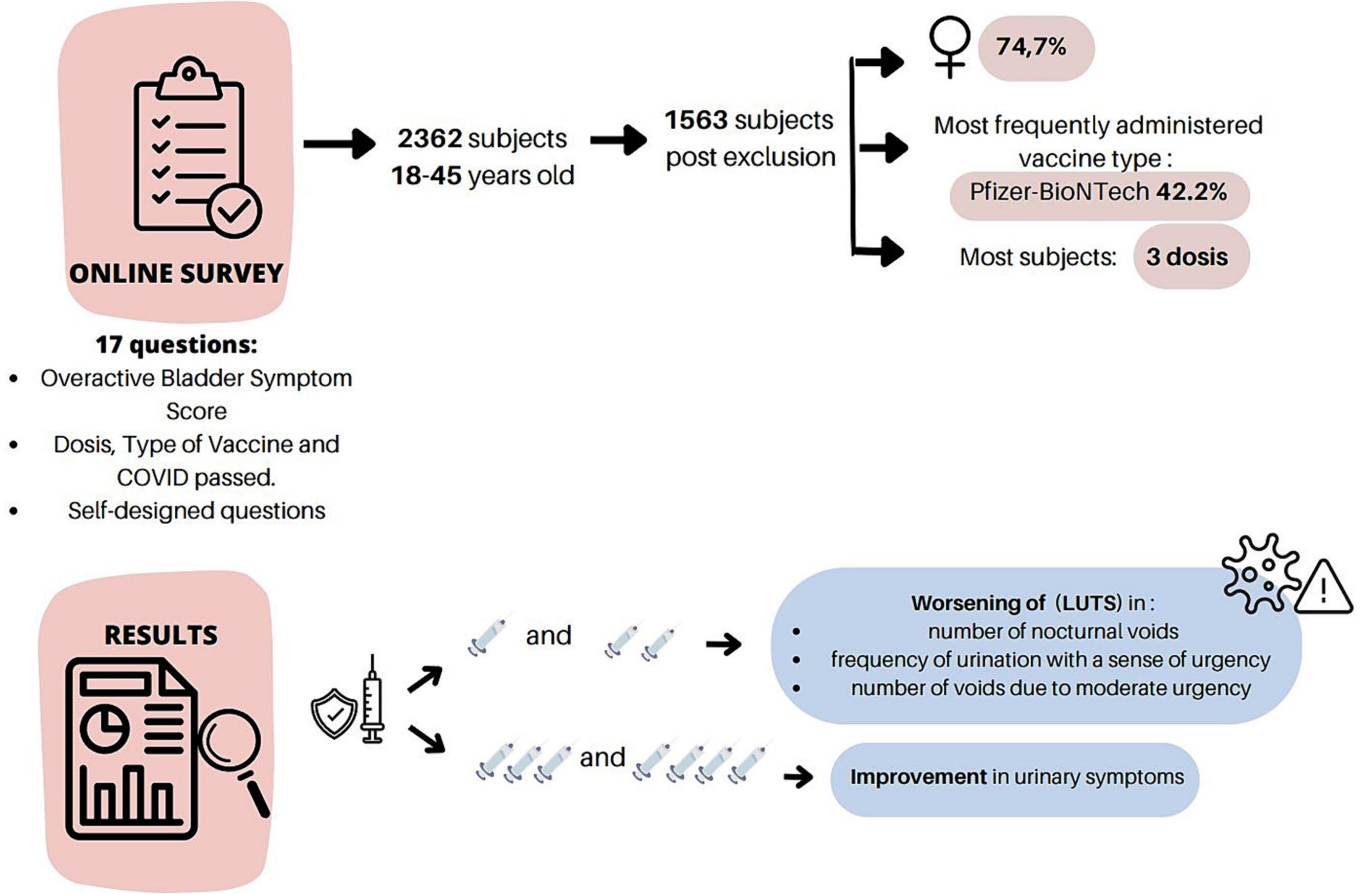
Scheme and development of the process.

No statistically significant differences were observed in terms of the frequency of not being able to reach the toilet in time before urination based on the number of vaccine doses received. However, depending on the participants’ sex, the percentage of men who have received one, two, and three doses of the vaccine and have *“never”* experienced urinary incontinence is significantly higher than that of women (83.3% vs. 56.7, 82.1% vs. 56.8, and 86% vs. 65.6%, respectively; *p* < 0.05). On the other hand, the percentage of women who have received one, two, and three doses of the vaccine and have *“rarely”* experienced urinary incontinence is significantly higher than that of men (33.3% vs. 10, 32.8% vs. 15, and 28.3% vs. 8.7%, respectively; *p* < 0.05). Additionally, the proportion of women who have received two doses of the vaccine and have experienced urinary incontinence “a few times per month” and “a few times per week” is significantly higher than that of men (5.8% vs. 1.7% and 4% vs. 0.6%, respectively; *p* < 0.05) (Figure 1).

## 4 Discussion

Although the journey toward global immunity against COVID-19 is not yet complete, in March 2023, at the time this study was conducted, 71.3% of the world’s population had already been vaccinated with at least one dose of the vaccine. In the case of Europe, the figure stood at 76%, and in Spain, it exceeds 85% of the population with complete vaccination (17).

Vaccination has been shown to prevent severe forms of the disease, hospitalization, death, and persistent COVID 19 (17). However, over these past 3 years, side effects of the different vaccines have been reported with varied symptoms, ranging from local symptoms at the injection site such as pain or stiffness, to general discomfort, fatigue, or weakness (7, 9).

Recent studies have discussed the effects of the vaccine on the urinary system, highlighting that the most common alterations are associated with urgency, frequency, nocturia, and urinary incontinence, all of which are consistent with the presence of an overactive bladder (OAB) (9). In this regard, the study conducted by Chen et al. (9) provides evidence of an association between overactive bladder (OAB) and COVID vaccination in the elderly population. Their findings indicate a worsening of lower urinary tract symptoms (LUTS) and an increase in the Overactive Bladder Symptom Score (OABSS) after the first dose of the vaccine, which aligns with the results reported by Zhao et al. (18, 19).

The present study, to the best of our knowledge, is the first to evaluate the long-term effect of COVID-19 vaccination on the deterioration of lower urinary tract symptoms (LUTS) and the Spanish version Overactive Bladder Symptom Score (OABSS-S), comparing the results among individuals who have received one, two, three, or four doses of vaccination.

Current evidence on overactive bladder demonstrates a relationship between OAB and anatomical and physiological changes associated with aging. Previous studies have indicated alterations in urinary frequency as adverse effects of COVID vaccines, but only in individuals aged 45 years and older, not in those younger than 45 (20). Therefore, this study aims to analyze the presence of overactive bladder in a younger population (18–45 years old) following COVID-19 vaccination. This population is considered to have a lower risk of lower urinary tract alterations and specifically, overactive bladder (OAB). In the results obtained in this study, regarding the OABSS-S questionnaire, a high frequency of daytime urination (“more than once every hour” - “every 1–2 h”) was observed in this young population, which was consistent across all types of vaccines: AstraZeneca (38.9%), Moderna (45.9%), Pfizer-BioNTech (43.8%), and the combination of these (36%).

Out of the 1,563 subjects analyzed, 90 (5.8%) received a single dose, 579 (37.0%) received two doses, 797 (51%) received three doses, and 97 (6.2%) received four doses. These findings align with the results reported by Chen et al. (9), after the first and second doses of vaccination, there was a worsening of lower urinary tract symptoms (LUTS) in terms of the number of nocturnal voids, frequency of urination with a sense of urgency, and number of voids due to moderate urgency. However, the results showed an improvement in urinary symptoms in subjects who received the third and fourth doses. The effects observed in this study can be explained through several mechanisms such as the immune response generated by the COVID-19 vaccine and its impact on the urinary tract. C-A Siegrist supports the idea that inflammation caused by cytokines could affect the smooth muscle of the bladder, decreasing the contractility threshold of the detrusor muscle, (21). But the observed effects could also be due to hormonal changes and immune-mediated reactions to vaccination, in addition to other factors not yet identified (22).

The present study demonstrates significant differences in how the adverse effects of vaccination can generate different responses in the urinary tract of men and women. Similarly, there are scientific publications that associate changes in the menstrual cycle of women after vaccination (23). Sex should be taken into account as a variable in the effects of the COVID-19 vaccine and its impact on the urinary tract. In the total sample, 74.7% were women, who had received more doses and a greater combination of different vaccines compared to men. The results show significant differences in the number of nocturnal voids in the questionnaire (OABSS-S), as men vaccinated with AstraZeneca have higher values compared to women who received the same vaccine (59.1% vs. 33.3%; *p* < 0.05). These findings are particularly noteworthy since the prevalence of nocturia in unvaccinated individuals is usually higher in young women than in young men, and the analyzed population is under 45 years old (24). Nocturia is also present in 33.7% of respondents vaccinated with Moderna and 29.2% of those vaccinated with Pfizer-BioNTech, who wake up once during the night to go to the bathroom. On the other hand, the proportion of men who received a combination of vaccines and have to urinate more than five times during the night is significantly higher than in women (1.7% vs. 0.2%; *p* < 0.05).

Regarding daytime urination, it is women who experience more symptoms after vaccination. Generally, up to seven episodes of urination during the day are considered normal, meaning that the bladder can hold urine for 3 or 4 h between bathroom visits (24). Women vaccinated with Moderna have reported that the time they can wait before urinating is only between 10 and 30 min, while for men, it was over 60 min. Women have lower values than men with the same vaccine (31.1% vs. 16.1%; *p* < 0.05) regarding the “Time they can hold before urinating.” Additionally, it is women who have experienced an inability to reach the toilet in time before urinating, leading to urinary leakage. On the other hand, men vaccinated with Moderna, Pfizer, or a combination of vaccines indicate that they have “never” experienced urinary leakage before reaching the bathroom.

Urge incontinence is a type of incontinence characterized by a sudden and strong urge to urinate, which leads to involuntary urine leakage (25). In this study, no statistically significant differences were observed in the sensation of daytime urinary urgency between men and women.

It is important to note that this study did not take into account other factors that could contribute to increased urinary frequency in women, such as pelvic floor dysfunction, pregnancy, postpartum period, hygiene habits, lifestyle modifications, dietary changes, or the intake of irritants such as caffeine or tobacco. These factors can indeed play a role in urinary symptoms and should be considered in future research (25). Absolutely, conducting studies that consider these factors is crucial for a comprehensive understanding of urinary symptoms and their relationship with COVID-19 vaccination.

## 5 Conclusion

COVID-19 vaccination has side effects on the lower urinary tract (LUT) and overactive bladder (OAB). The results show that after the first dose of the vaccine, LUT symptoms worsened, and OABSS scores increased in subjects under the age of 45. However, symptoms improved after the third and fourth doses.

The study also revealed differences in the effects of vaccination between men and women. Changes in daytime and nighttime urinary frequency were observed, with a higher number of nocturnal voids in men vaccinated with AstraZeneca and more daytime voids in women vaccinated with Moderna. However, no differences were found in the sensation of daytime urinary urgency between both sexes.

Based on the results obtained in this study, monitoring and addressing urinary tract side effects of COVID-19 vaccination are important for vaccination programs to include the systematic collection of urinary tract side effect data, which will allow for ongoing evaluation of vaccine safety and efficacy. Health care professionals should be alert to the possibility of patients experiencing urinary tract symptoms following COVID-19 vaccination.

## 6 Limitations

The self-reported nature of the survey data may introduce biases and limitations in the interpretation of the results. It is important to consider that this study focused specifically on a population aged 18 to 45 years, which limits the ability to extrapolate the results to different age groups. Further studies with a rigorous design are needed to evaluate the immune response in patients after multiple doses of vaccination.

## 7 Future research

Additional research is needed to understand the mechanisms underlying the observed urinary tract effects after COVID-19 vaccination.

Longitudinal studies that follow participants over time are needed to assess changes in urinary tract symptoms before and after vaccination and expand our understanding of the effects of COVID-19 vaccination.

## Data availability statement

The original contributions presented in the study are included in the article/Supplementary material, further inquiries can be directed to the corresponding author.

## Ethics statement

The studies involving humans were approved by Ethics Committee of University European of Madrid (reference number: CIPI/22.247). The studies were conducted in accordance with the local legislation and institutional requirements. The participants provided their written informed consent to participate in this study. Written informed consent was obtained from the individual(s) for the publication of any potentially identifiable images or data included in this article.

## Author contributions

Md-l-P-S-F: Data curation, Investigation, Methodology, Project administration, Visualization, Writing – original draft. EG-G: Formal analysis, Investigation, Methodology, Writing – original draft. BM-P: Conceptualization, Formal analysis, Supervision, Writing – original draft. IE: Methodology, Software, Writing – original draft. DD-B: Conceptualization, Supervision, Writing – original draft. MS-R: Data curation, Formal analysis, Investigation, Writing – original draft.
