# Supplementary material for: Effects of vaccination against COVID-19 on overactive bladder symptoms on young population
Source: Front Med (Lausanne). 2024 Jun 25;11:1338317. doi: 10.3389/fmed.2024.1338317 (PMC11231098; doi:10.3389/fmed.2024.1338317)
Supplement: Supplementary file 1 [file Data_Sheet_1.pdf]

APPENDIX 1.

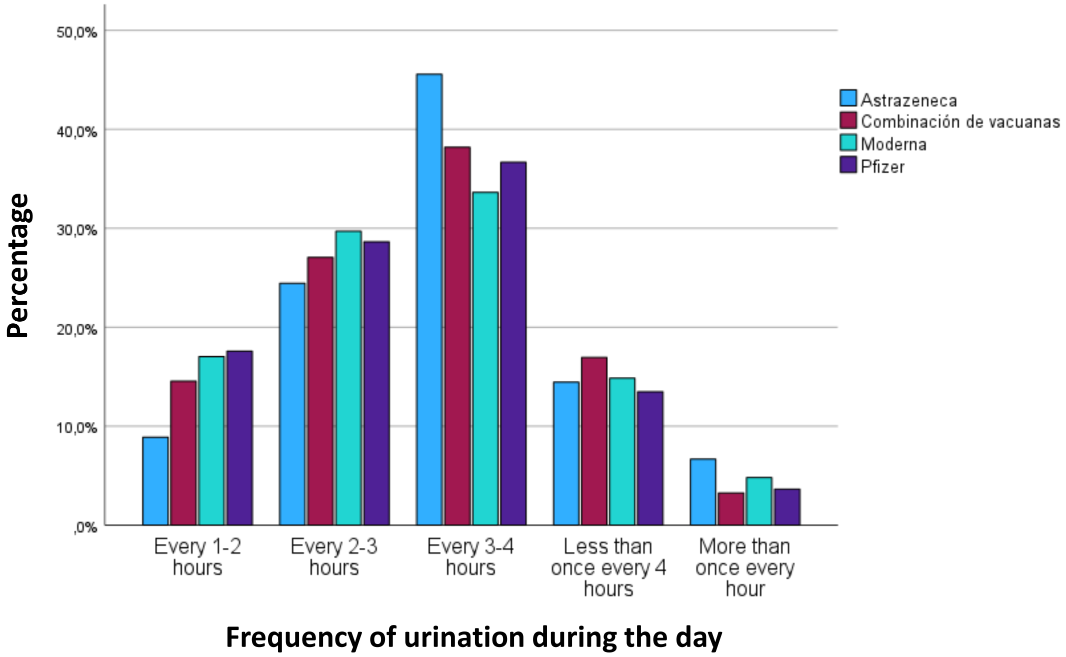

Figure 1. Results of frequency of urination during the day depending on the type of vaccine

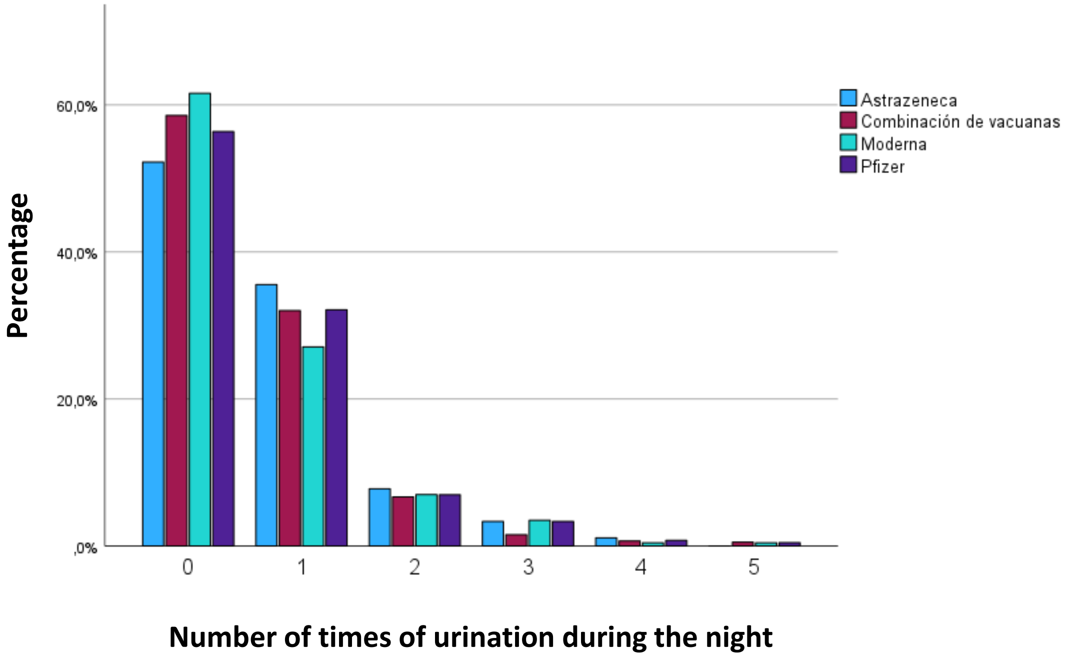

Figure 2. Results of numbers of times of urination during the night depending on the type of vaccine

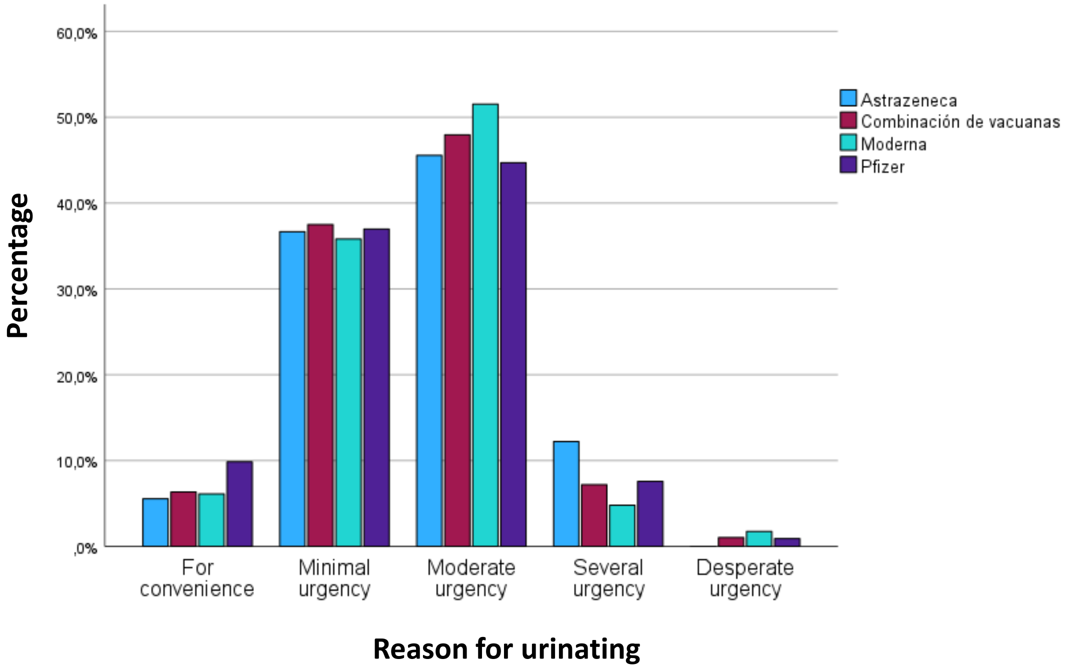

Figure 3. Results of reason for urinating depending on the type of vaccine

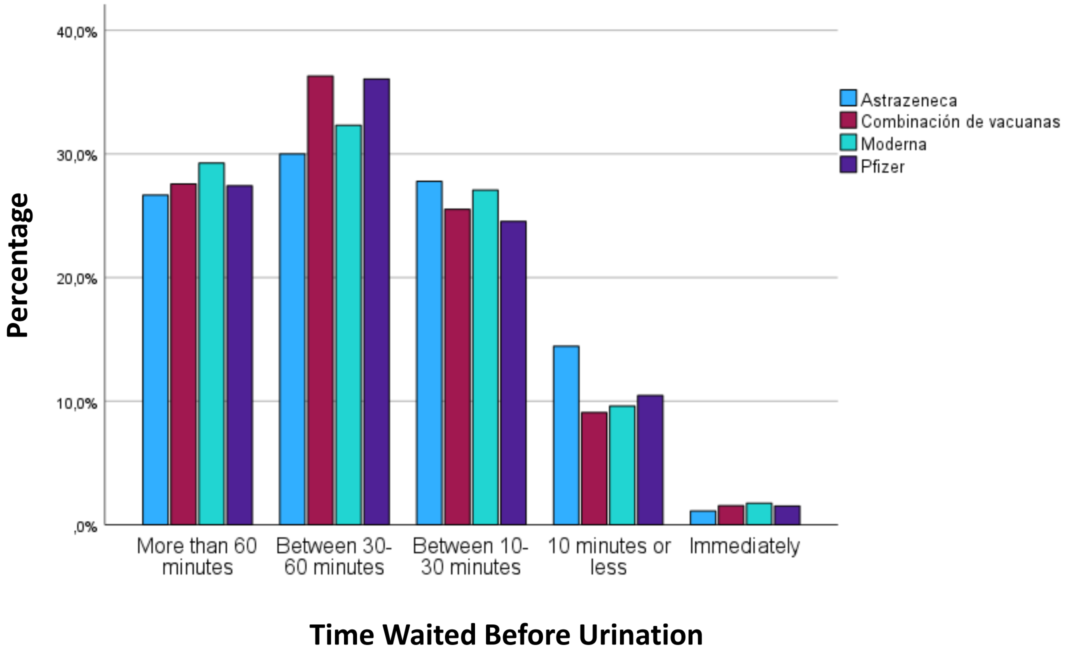

Figure 4. Results of time waited before urination depending on the type of vaccine

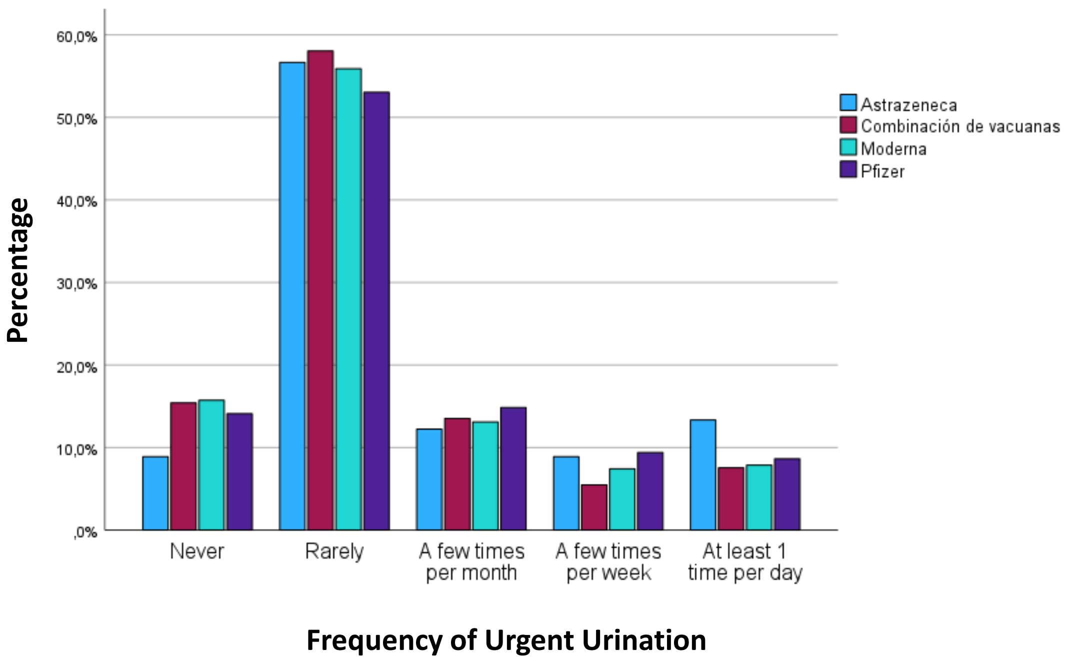

Figure 5. Results of frequency of urgent urination depending on the type of vaccine

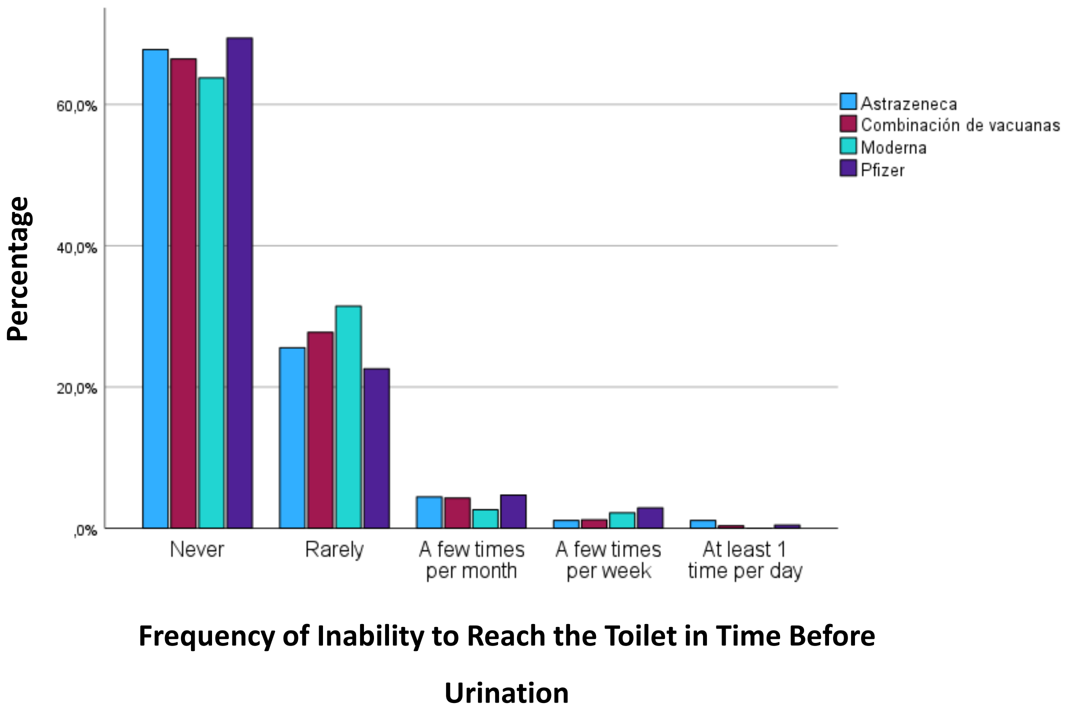

Figure 6. Results of frequency of inability to reach the toilet in time before urination depending on the type of vaccine
